# Humero‐anconeal elbow incongruity in spaniel breed dogs with humeral intracondylar fissure: Arthroscopic findings

**DOI:** 10.1111/vsu.13728

**Published:** 2021-09-28

**Authors:** Alan Danielski, Russell Yeadon

**Affiliations:** ^1^ The Ralph Veterinary Referral Centre Marlow UK; ^2^ Department of Veterinary Medicine and Animal Sciences University of Naples “Federico II” Naples Italy; ^3^ Lumbry Park Alton UK

## Abstract

**Objective:**

To report arthroscopic findings in dogs with humeral intracondylar fissure (HIF) and compare these findings in joints of dogs not affected by HIF on preoperative CT images.

**Study design:**

Controlled clinical study.

**Animals:**

Dogs with HIF (14 dogs, 21 elbows) and dogs without HIF (20 dogs, 31 elbows).

**Methods:**

A caudo‐medial arthroscope portal was used to inspect all elbow joints. Arthroscopic features of 21 joints of dogs with HIF were compared with 31 control elbows of HIF‐ negative dogs.

**Results:**

All elbows with HIF showed a focal cartilage lesion on the caudal aspect of the humeral condyle. The lesions ranged from a simple indentation into the articular surface to a full thickness cartilage erosion. Humero‐anconeal incongruity was identified in all elbows with HIF as absence of joint space at the point of contact between the tip of the anconeal process and the cartilage lesion, with a wider joint space distally within the ulnar trochlear notch. None of the elbows without HIF showed the cartilage lesion or evidence of humero‐anconeal incongruity.

**Conclusion:**

Use of a novel arthroscope portal allowed description of a previously unreported cartilage lesion on the caudal humeral condyle of dogs with HIF. The lesion was found in all dogs with HIF but in no dogs without HIF.

**Clinical significance:**

Humero‐anconeal incongruity and an associated cartilage lesion appear to be present in dogs with HIF. We propose that this lesion may be associated with humero‐anconeal incongruity. This may be considered as a possible future therapeutic target for HIF.

**Abbreviations**
CT: computed tomography
HA lesion: humero‐anconeal lesion
HIF: humeral intracondylar fissure
IOHC: incomplete ossification of the humeral condyle

## 1 INTRODUCTION

Humeral intracondylar fissure (HIF) is a recognized cause of thoracic limb lameness in spaniel breed dogs.^1^, ^2^, ^3^ Initial suggestions regarding etiopathogenesis involved failure of the fusion of the two centers of ossification of the humeral condyle (incomplete ossification of the humeral condyle; IOHC).^3^ More recently, this theory was challenged by a study that reported the development of a fissure in the humeral condyle of a 5‐year‐old Cocker Spaniel that 2 years previously had a completely normal elbow on computed tomography (CT).^4^ A similar case report documented propagation of a partial fissure of the humeral condyle in an American Cocker Spaniel, most likely due to abnormal joint biomechanics.^5^ The findings of these cases were suggested to support a previously noted hypothesis that stress fracturing may be involved in formation of humeral intracondylar fissures or incomplete fractures.^6^ An electron microscopy study on failure mode of transcondylar screws further supported the role of intracondylar instability due to abnormal elbow joint biomechanical forces that might be associated with HIF.^7^ However, precise etiopathogenesis of HIF has yet to be elucidated.

The objective of this descriptive study was to report a previously undocumented cartilaginous lesion, henceforth referred to as a humero‐anconeal lesion (HA lesion) throughout the text, present on the caudal aspect of the medial humeral condyle of 21 elbows with HIF, which the authors believe may indicate impingement between anconeal process and humeral condyle. Their hypothesis was that this lesion would be present in elbows with CT‐confirmed HIF, and would not be found in elbows without HIF.

## 2 MATERIAL AND METHODS

Dogs presenting to the authors’ institution between September 2019 and September 2020 for unilateral or bilateral thoracic limb lameness and signs of pain on elbow manipulation were included in this study. Dogs not undergoing both CT and elbow arthroscopy were excluded. Elbows were subsequently divided into two groups depending on the presence or absence of HIF on CT imaging. The groups were termed “HIF positive” and “HIF negative.”

Computed tomography (GE Revolution, GE Healthcare, Chalfont St Giles, UK) of both thoracic limbs from the carpi to the shoulders was performed preoperatively with the dog under deep sedation (3‐8 mcg/kg dexmedetomidine and 0.2 mg/kg butorphanol, IV). Dogs were positioned in sternal recumbency, with the elbow joints parallel and extended cranially at approximately 130°‐140° of extension. If CT revealed changes of the humeral condyle compatible with development or presence of HIF (as previously described by Carrera et al.),^8^ the affected elbow was included in the HIF positive group. All remaining cases that showed no evidence of HIF on preoperative CT examination but where there were other bone changes suggestive of joint pathology (such as fragmented coronoid process, kissing lesions, and medial compartment disease) were included in the HIF negative group.

### 2.1 Arthroscopic technique

**FIGURE 1:**
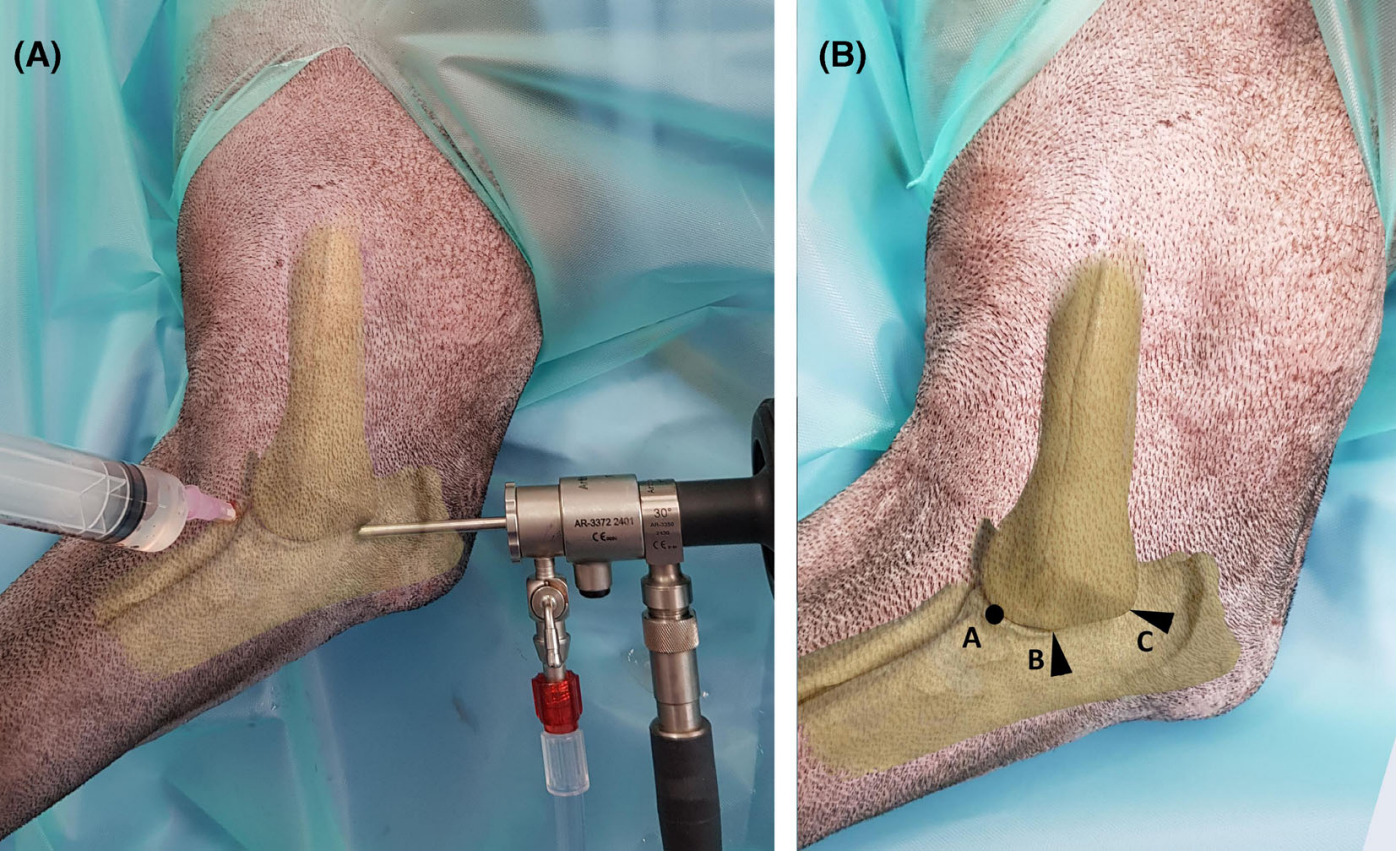
(A) Medial view of right elbow with bone model overlay showing the positioning of the egress and arthroscope portals. (B) Medial view of right elbow with bone model overlay showing annotated positioning of the instruments for joint arthroscopy (circle “A”: egress portal; closed arrow “B”: traditional medial location and entry orientation of the arthroscope portal as described by Beale et al.;^9^ closed arrow “C”: “novel” caudo‐medial location and entry orientation of the arthroscope portal used in this study)

**FIGURE 2:**
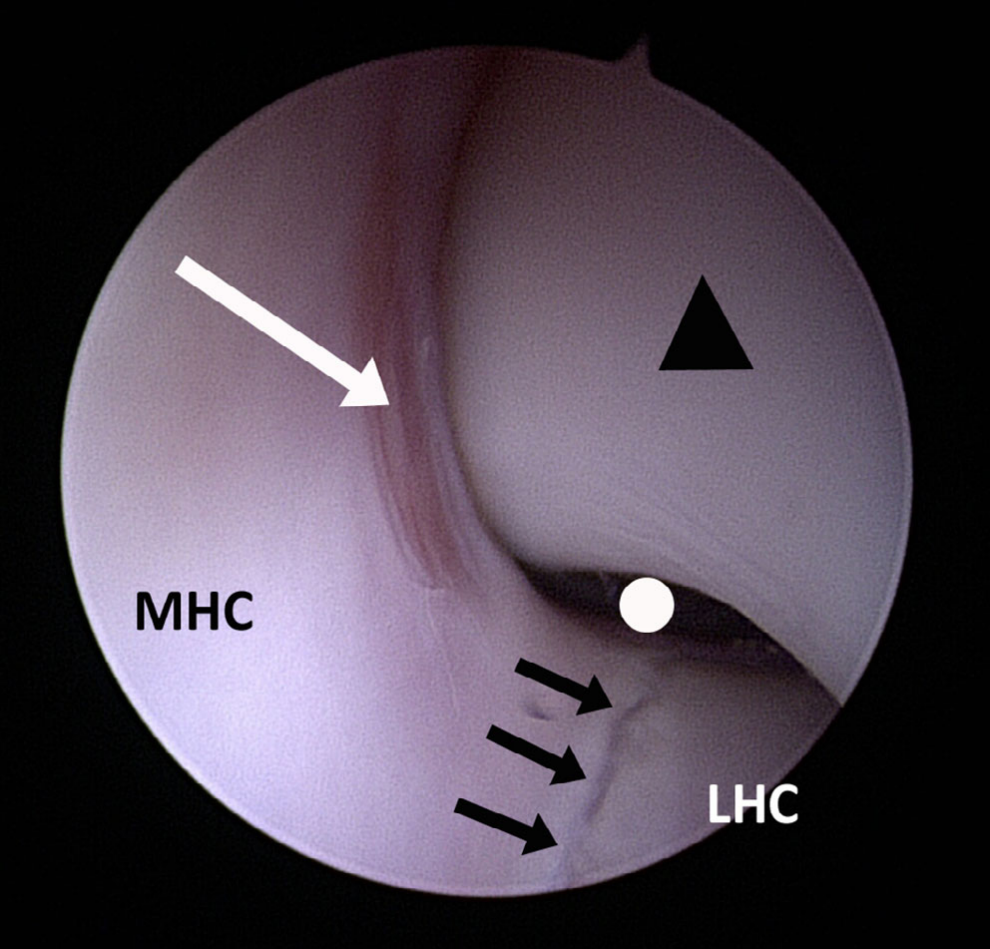
Intra‐articular structures of the caudal aspect of the right elbow joint as seen through the caudo‐medial arthroscopic portal. Black triangle: medial aspect of the anconeal process; white arrow: humero‐anconeal cartilage lesion; white circle: olecranon fossa; black arrows: humeral intracondylar fissure; MHC: medial humeral condyle; LHC: lateral humeral condyle

Elbow arthroscopy, using a 2.4 mm, 30° oblique arthroscope (Arthrex, Munich, Germany), was performed with the dog under general anesthesia in dorsal recumbency. A sand bag was positioned adjacent to the affected elbow, to be used as a fulcrum for initial joint distraction. The skin was aseptically prepared and the limb draped according to standard surgical technique. A surgical assistant held the distal limb such that the humerus was parallel to the floor with the sandbag positioned under the distal humerus to help as a fulcrum. The distal extremity was concomitantly externally rotated (pronated) to allow additional opening of the medial aspect of the joint space (Figure 1A). The egress portal (Figure 1B, label “A”) was established first by inserting an 18 gauge needle, connected to a 10 mL syringe filled with sterile lactated Ringer's solution, cranio‐medially to the elbow joint, aiming just caudal to the medial collateral ligament and to the median nerve in a caudo‐lateral direction. Once synovial fluid was aspirated to confirm intra‐articular placement, the sterile Ringer's solution was instilled to distend the joint. The arthroscope portal was established next by making a small skin incision with a no. 11 blade and inserting the arthroscope cannula with the attached blunt obturator caudal to the medial epicondyle and the ulnar nerve, in a cranio‐lateral direction, parallel to the medial surface of the anconeal process of the ulna (Figure 1B, label “C”). The arthroscope portal was located midway between the most prominent part of the medial epicondyle of the humerus and the olecranon tuber of the ulna, being more caudally located than that previously reported (Figure 1B, label “B”).^9^ Typically the skin incision would be made along the palpable and visible line of indentation between the cranial border of the triceps muscle and the caudal aspect of the medial humeral epicondyle, taking care to avoid the ulnar nerve in this region. The following structures were arthroscopically inspected: medial aspect of the coronoid process of the ulna, radial head, medial collateral ligament, insertion of the biceps brachii muscle adjacent to the medial aspect of the coronoid process of the ulna, lateral aspect of the coronoid process of the ulna, medial humeral condyle, medial aspect of the ulnar notch up dorsally to include the anconeal process, medial humeral condyle up to the olecranon fossa, and approximately the medial third of the lateral humeral condyle. Particular attention was given to the area where the anconeal process was engaging the humeral condyle at the caudal aspect of the humero‐ulnar articulation (Figure 2).

During arthroscopy, the limb was manipulated throughout a range of motion from maximal extension to approximately 120°‐140° of flexion to assess if the anconeal process was impinging the humeral condyle and if there were signs of dynamic incongruency, such as if a steplike clunk could be felt and to evaluate for variations in humero‐ulnar joint space width, particularly in the area of the tip of the anconeal process and olecranon fossa. During limb manipulation, no pronation or supination force was applied to the distal limb, with the exception of gentle pronation with the elbow held in approximately 120°‐140° of flexion to allow subjective evaluation of joint congruity during this manipulation.

Presence of fragmentation or fissure formation of the medial aspect of the coronoid process of the ulna was recorded, as was the severity and location of any cartilage damage present, including use of the previously described modified Outerbridge classification system.^10^

### 2.2 Statistical analysis

Fisher's exact test was used to test the incidence of presence or absence of the HA lesion in the HIF positive and HIF negative groups. The test was repeated including only spaniel breed dogs in the HIF negative group to explore the possibility of breed as a confounding variable. *P*‐values <.05 were considered to be startistically significant.

## 3 RESULTS

The HIF positive group consisted of 21 elbows (14 dogs). These dogs (9 English Springer Spaniels and 5 Cocker Spaniels) had a median age of 6 years 1 month (range 9‐101 months); 11 were males (5 neutered) and 3 were neutered females. All dogs presented with signs of lameness of at least one thoracic limb, and all had pain on elbow joint manipulation, particularly in elbow extension. Computed tomography of these elbows revealed presence of complete hypoattenuating (*n* = 11) or incomplete hypoattenuating (*n* = 10) lines between the medial and lateral condyle and presence of sclerosis adjacent to the hypoattenuating lines consistent with the previous description of HIF by Carrera et al.^8^

**FIGURE 3:**
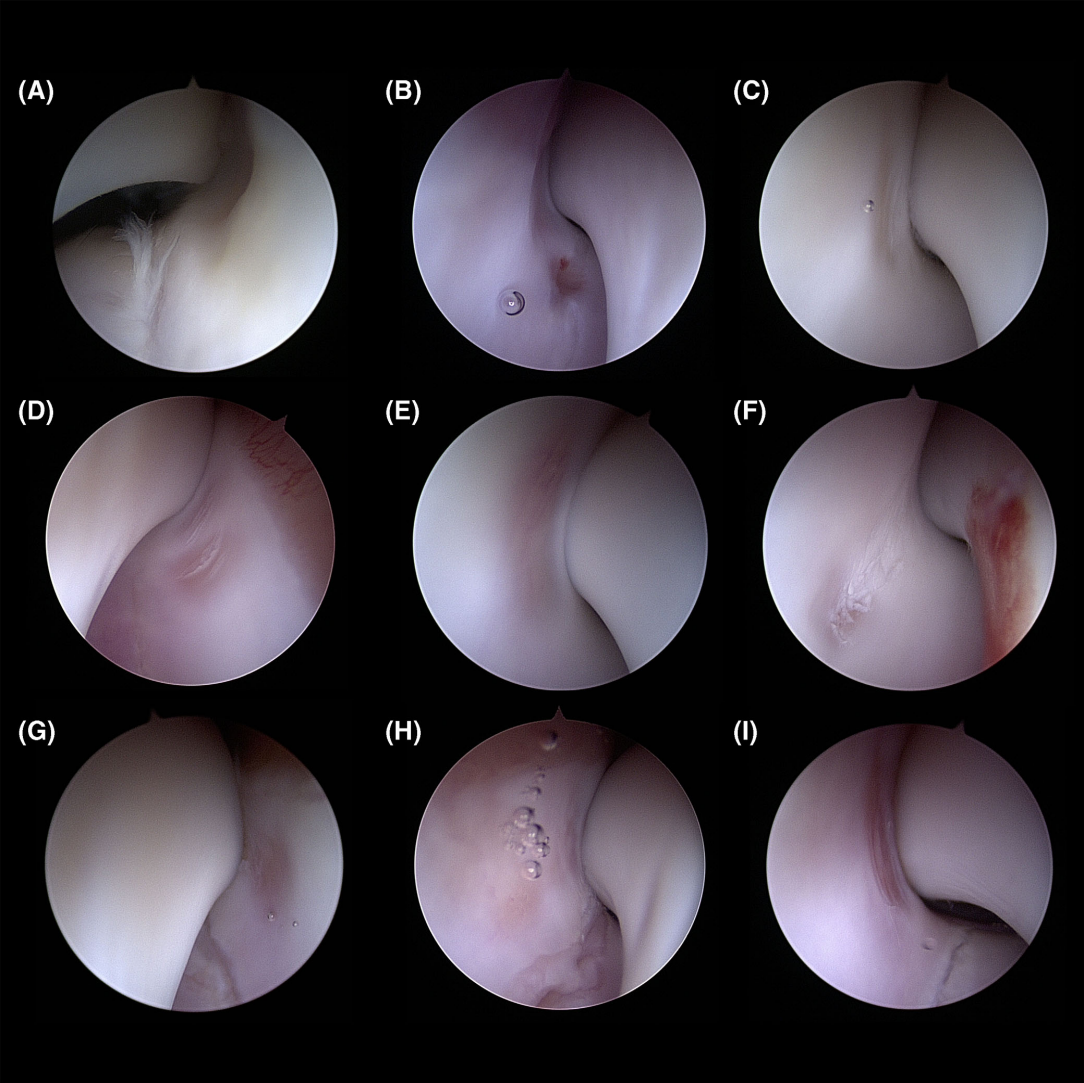
Different examples of HA lesions. (A, B) Indentation into the humeral cartilage but with cartilage appearance being grossly normal. (C) lesion with superficial cartilage fibrillation (modified Outerbridge grade II). (D–H) partial thickness cartilage abrasion (modified Outerbridge grade III). (I) full thickness cartilage abrasion with exposure of subchondral bone (modified Outerbridge grade IV). Images (A), (D) and (G) are of left elbows. Images (B), (C), (E), (F), (H) and (I) are of right elbows

All elbows in the HIF positive group showed an arthroscopically visible focal cartilage lesion (4‐10 mm in diameter) on the caudal aspect of the humeral condyle, immediately distal to the supratrochlear foramen, at its closest point, which was approximately 0.5‐3 mm medial to the “isthmus” of the humeral condyle (or, when visible, medial to the HIF line, which was evident in 19/21 elbows). This HA lesion was, variously, an indentation into the humeral cartilage but with cartilage appearance being grossly normal (*n* = 3; Figure 3A and B), a lesion with cartilage fibrillation (modified Outerbridge grade II, *n* = 4; Figure 3C), a partial thickness focal lesion (grade III, *n* = 13; Figure 3D–H), or a full thickness focal cartilage loss (modified Outerbridge grade IV, *n* = 1; Figure 3I). In most elbows the HA lesion appeared to be circular or elliptical in shape, but in 6/21 elbows the main focal lesion was accompanied by 1‐2 smaller lesions present in the immediate vicinity, typically immediately dorsal to the main lesion, presenting the same degree of cartilage damage of the main lesion. In all cases, when the elbow was extended to 120°‐140°, the tip of the anconeal process perfectly matched the HA lesion and in 16/21 elbows it was possible to feel a clunk‐like sensation when the extension‐flexion movement was repeated or when pronation was performed at this joint angle; this reproducibly coincided with the anconeal process visibly “dropping” into the indented lesion and “re‐emerging” from it. Precise additional data regarding incidence of this phenomenon were not recorded as consistent reproduction of this, even in a single elbow, was felt to be slightly variable and subjective in nature. In this circumstance, the humero‐ulnar joint space was always absent at the level of the tip of the anconeal process, and it was noticeably wider further distally along the ulnar notch. Subjectively, when the anconeal process was engaging the lesion and concomitant pronation and supination were applied, there appeared to be an opening of the HIF line by approximately 0.5 mm in 5/21 elbows, all of which were confirmed as having a complete hypoattenuating line on CT. Fragmentation of the medial aspect of the coronoid process of the ulna was concomitantly identified in 6/21 elbows but no elbow had any evidence of significant cartilage pathology (modified Outerbridge grade ≥ I) associated directly with this lesion or at any part of the cranial portion of the elbow joint.

The HIF negative group consisted of 31 elbows (20 dogs). Median age was 5 years 8 month (range 9‐141 months); 12 were male (7 neutered) and 8 female (7 neutered). Breed included Labrador Retrievers (8), cocker and English Springer Spaniels (4), cross breed (3) and 1 each of boxer, Staffordshire bull terrier, German shepherd, labradoodle and basset hound. Eleven elbows were diagnosed with fragmentation of the medial coronoid process, 4 had focal medial humeral condyle “kissing lesions” (representing areas of cartilage pathology of modified Outerbridge grade II or higher) and 5 with advanced medial compartment disease (representing areas of diffuse cartilage pathology of modified Outerbridge grade IV or V affecting both the cranial portion of the medial humeral condyle and the coronoid process of the ulna). Areas of cartilage pathology in all elbows were limited to the cranial portion of the elbow joint and did not extend into the caudal portion of the humero‐ulnar articulation.

No dog in the HIF negative group had any arthroscopic evidence of the HA lesion on the caudal aspect of the medial humeral condyle, with cartilage of normal visual appearance in the area of the HA lesion in the HIF positive group. When the joint was arthroscopically assessed and the elbow extended and flexed, the joint space between humerus and ulnar notch always appeared uniform, and maintained a consistent joint space width at the level of the tip of the anconeal process.

The incidence of the HA lesions was different between the HIF positive group and the HIF negative group (*P* value <.0001), and between the HIF positive group and HIF negative spaniel breed dogs (*P* value <.0001).

In all elbows, examination of all joint structures listed above was possible through the arthroscope portal location described.

## 4 DISCUSSION

A previously unreported cartilage lesion on the caudal aspect of the medial humeral condyle was identified in dogs with HIF and it was not present in a control group of unaffected dogs. Use of a caudo‐medial arthroscope portal, modified by comparison with previously published descriptions of elbow arthroscope portals, and specific joint manipulation during arthroscopy facilitated viewing of this lesion.

Several theories have been postulated to explain the etiopathogenesis of HIF.^3^, ^4^, ^5^, ^7^, ^11^ The incomplete ossification hypothesis, first developed in 1989, does not fit with the cohort of dogs in our study because of the delay in onset of clinical signs. In fact, in the veterinary literature, most case series documented a median age at presentation of approximately 3‐4 years (whereas age of onset for true incomplete ossification might be expected to be somewhat younger), and, in our study, this was supported by a median age at presentation of more than 6 years.^1^, ^2^, ^3^ Furthermore, at least 2 case reports have documented apparent development of HIF in elbows previously found to be HIF negative on CT.^4^, ^5^

A fatigue fracture hypothesis has also been proposed^4^, ^7^, ^12^ and, as a consequence, the terminology “HIF” has been preferred over the previous nomenclature of “incomplete ossification of the humeral condyle” or “IOHC.” The histopathology findings published by Marcellin‐Little in 1994 (presence of fibrous tissue, increased osteoclastic activity and increased plasma cells, surrounded by a border of osteosclerosis) might in fact be considered suggestive of, or at least consistent with, an adaptive response to persistent mechanical forces rather than being related to failed endochondral ossification.^3^, ^4^ Similarly, the histological features of a bone biopsy harvested from the fissure line of a pointer dog with HIF seemed to support the effect of an adaptive response to excessive and recurrent mechanical forces.^12^

Analysis of broken transcondylar screws published by Charles et al. in 2009 suggested that chronic multiplanar intracondylar instability might result in subsequent fatigue failure of transcondylar implants at the level of the intracondylar fissure.^7^ It was postulated that there could be the potential for the condyles to be “driven apart” by the wedge‐shaped proximal ulna resulting in fatigue failure of the screw.^7^ Our arthroscopic findings suggest that mechanical impingement of the anconeal process within the olecranon fossa, as evidenced both by presence of the cartilage lesion and the apparent humero‐anconeal joint space narrowing on arthroscopy in our HIF‐positive group might better account for this predicted intracondylar instability. By comparison, we would postulate that the “wedge theory” might be expected to produce a more diffuse pattern or cartilage pathology along the ulnar trochlear notch and/or the opposing portion of the humeral condyle.

In 5 HIF‐positive elbows, widening of the HIF line was identified arthroscopically when the tip of the anconeal process was engaged into the HA lesion and pronation/supination was applied, supporting an association between these lesions. The presence of the HA lesion only at the medial aspect of the HIF line, with no abnormalities of the lateral humeral condyle seen, along with the palpable “clunk” or a visual “flick” of the image when the tip of the anconeal process was engaged into and out of the HA lesion in 16/21 elbows, suggests that these lesions are likely to be associated with a focal incongruity, making the broad “humero‐ulnar incongruency” terminology being considered a much less probable explanation. We therefore propose that a terminology of “humero‐anconeal incongruity” might be considered to describe these findings.

In 10 of 21 HIF‐positive elbows, preoperative CT imaging revealed that an incomplete HIF line was present. In these cases, the fissure was subjectively considered to be most evident at the most caudo‐dorsal aspect of the humeral condyle but not at the most cranial aspect. This finding, that the HIF line appears to start caudally and may then progress further cranially, could perhaps also be consistent with our theory that HIF may be a consequence of “humero‐anconeal incongruity” affecting the caudal portion of the joint. However, this cannot be confirmed in the absence of sequential CT studies of the same cases or further biomechanical data, which may be subjects for further study. A similar pattern of fissure propagation was demonstrated whilst investigating the etiopathogenesis of fragmentation of the medial aspect of the coronoid process of the ulna.^13^ Radio‐ulnar incongruency caused the greatest amount of fatigue microdamage to the subchondral bone in the proximity of the area where the radial head and coronoid process were in conflict but also, to a lesser extent, to the adjacent areas. A similar fatigue fracture theory could potentially be applied to the development of HIF with humero‐anconeal incongruency instigating a fissure at the caudal aspect of the humeral condyle and subsequently progressing cranially. Our observation of the HA lesion in elbows with an incomplete HIF line on CT further supports the theory that humero‐anconeal incongruity may precede HIF development, although the precise sequence and etiopathogenesis of development of these features cannot be definitively confirmed from our study. It is worth noting that no elbow in our HIF‐negative group had any evidence of the HA lesion, and that this group included four spaniel breed dogs (seven elbows), therefore suggesting that the HA lesion is directly associated with HIF development and is not likely to be a coincidental breed‐related variant or incidental finding. Our statistical analysis supports the view that the HA lesion appears to be associated with HIF and does not appear to be a breed‐dependent finding. Further study is warranted to establish whether early identification of humero‐anconeal incongruity or the HA lesion might be viable as a potential screening modality in spaniel breed dogs to help predict future HIF development, or whether it might reliably facilitate diagnosis of HIF as an isolated diagnostic test where CT is not available.

The principal imaging tools currently used to assess elbow incongruity are radiography, computed tomography and arthroscopy. Radiography remains a common screening method because it is cost effective and widely available but it is known to be an insensitive means of evaluating elbow joint incongruity accurately, particularly where the incongruity is relatively modest in nature.^14^, ^15^, ^16^ In recent years, CT has become a more popular technique to detect incongruity, proving to be a very accurate method for assessing the joint space.^17^, ^18^, ^19^ However, a more recent study showed that radio‐ulnar congruency, assessed by the evaluation of CT images, is strongly influenced by elbow position with regards to the maximal pronation or supination of the elbow joint, confounding the diagnosis of elbow incongruency.^20^ Arthroscopy has been reported to be 94% to 98% sensitive and 81% to 89% specific in detecting elbow incongruency and cartilage changes.^21^, ^22^, ^23^ Arthroscopy provides magnification and illumination and it therefore enhances observation and identification of cartilage pathology and, as in this case series, of dynamic incongruency, which cannot be assessed with other static diagnostic tools. However, it is of note that the evaluation of dynamic elbow incongruity in clinical cases remains somewhat subjective and a single optimal protocol in terms of joint manipulation with arthroscopic guidance has not been established. The difference in joint space between humerus and ulnar notch can therefore be labeled as a potentially subjective finding, although it is felt that the identification of an HA lesion is a reliable consistent feature.

The modification of the previously reported arthroscope portal to a caudo‐medial location allowed the inspection of all major intra‐articular structures of the medial elbow joint compartment and partially of the lateral compartment that would usually be assessed through the traditional medial arthroscope portal. However, this caudo‐medial portal also allowed the caudal (in some elbows even the caudo‐dorsal) aspect of the humeral condyle to be inspected, permitting the identification of the HIF focal cartilage lesion. This caudal portal is routinely used at the authors’ institution for all elbow arthroscopy cases. Subjectively, this portal is noninferior to the previously reported arthroscope portal location in terms of ability to evaluate common elbow joint pathologies including those associated with the medial aspect of the coronoid process, the insertion of the biceps brachii tendon, the cranial portion of the medial humeral condyle, the radial head, and the anconeal process of the ulna. There may be an additional benefit in some situations, such as where extensive osteophytosis may make joint evaluation through the previously described arthroscope portal more challenging, although it falls beyond the scope of this study to evaluate or report this further. In this study, the new portal was not associated with any obvious iatrogenic cartilage damage to the weight‐bearing articular surface of medial humeral condyle and ulna, even throughout joint manipulation. When the latter was performed, joint visualization with the arthroscope was possible from when the joint was fully extended to when it was flexed to approximately 45°. At flexion angles of <40°, joint examination was typically impossible due to periarticular soft tissues extending over the tip of the arthroscope, and with a tendency for the arthroscope to become displaced out of the joint space. It is not known whether the learning curve for this caudo‐medial arthroscope portal might differ from that of the conventional medial arthroscopic portal, or whether iatrogenic cartilage damage, or damage to the arthroscope itself, or to periarticular structures such as the ulnar nerve might be more likely when performed by less experienced arthroscopists, particularly when combined with intraoperative joint manipulation. This might be an area for further study.

Two major limitations of this observational study are the small case number and the overrepresentation of spaniel breed dogs, although the statistical findings of Fisher's exact testing suggest that these were relatively minor limitations. Other limitations include the lack of elbows free of any form of pathology as a control group and the fact that the study was not blinded, which could have introduce further bias to the results.

It is widely accepted that once formed, HIFs do not tend to progress to bone union, even after placement of a transcondylar screw.^8^ This would support the theory of ongoing low‐grade intracondylar instability, and the evidence of humero‐anconeal incongruity provided here could be a further potential explanation to account for this, in addition to those hypotheses previously postulated. If an etiopathogenic link between humero‐anconeal incongruity and HIF can be confirmed, further evaluation of alternative treatment modalities intended to address joint incongruity may play a future role in the treatment of HIF and warrant further study.

## CONFLICT OF INTEREST

The authors declare no conflicts of interest related to this report

## AUTHOR CONTRIBUTIONS

Danielski A, DVM DECVS: Conception of study, study design, literature review, acquisition of data, data analysis and interpretation, drafting, revising, and approval of the submitted article. Yeadon R, MA VetMB, CertSAS, DECVS: study design, literature review, acquisition of data, data analysis and interpretation, drafting, revising and approval of the submitted article.

## Supporting information


**Video S1.** Supporting information

## References

[1] Moores AP , Agthe P , Schaafsma IA . Prevalence of incomplete ossification of the humeral condyle and other abnormalities of the elbow in English Springer Spaniels. Vet Comp Orthop Traumatol. 2012;25(3):211‐216. 22286098 10.3415/VCOT-11-05-0066

[2] Moores AP , Moores AL . The natural history of humeral intracondylar fissure: an observational study of 30 dogs. J Small Anim Pract. 2017;58(6):337‐341. 28369948 10.1111/jsap.12670

[3] Marcellin‐Little DJ , DeYoung DJ , Ferris KK . Incomplete ossification of the humeral condyle in Spaniels. Vet Surg. 1994;23:475‐487. 7871711 10.1111/j.1532-950x.1994.tb00509.x

[4] Farrell M , Trevail T , Marshall W , Yeadon R , Carmichael S . Computed tomographic documentation of the natural progression of humeral intracondylar fissure in a Cocker Spaniel. Vet Surg. 2011;40(8):966‐971. 22091831 10.1111/j.1532-950X.2011.00906.x

[5] Witte PG , Bush MA , Scott HW . Propagation of a partial incomplete ossification of the humeral condyle in an American Cocker Spaniel. J Small Anim Pract. 2010;51(11):591‐593. 20973787 10.1111/j.1748-5827.2010.00988.x

[6] Butterworth SJ , Innes JF . Incomplete humeral condylar fractures in the dog. Journal of Small Animal Practice. 2001;42(8):394–398. doi:10.1111/j.1748-5827.2001.tb02488.x 11518419

[7] Charles EA , Ness MG , Yeadon R . Failure mode of transcondylar screws used for treatment of incomplete ossification of the humeral condyle in 5 dogs. Vet Surg. 2009;38:185‐191. 19236676 10.1111/j.1532-950X.2008.00486.x

[8] Carrera I , Hammond GJ , Sullivan M . Computed tomographic features of incomplete ossification of the canine humeral condyle. Vet Surg. 2008;37(3):226‐231. 18394068 10.1111/j.1532-950X.2008.00370.x

[9] Beale BS , Hulse DA , Schulz KS , et al. Small Animal Arthroscopy. Saunders; 2003:51‐79.

[10] Farrell M , Heller J , Solano M , Fitzpatrick N , Sparrow T , Kowaleski M . Does radiographic arthrosis correlate with cartilage pathology in Labrador Retrievers affected by medial coronoid process disease? Vet Surg. 2014;43(2):155‐165. 24393075 10.1111/j.1532-950X.2014.12092.x

[11] Sjostrom L , Kasstrom H , Kollberg M . Ununited anconeal process in the dog: pathogenesis and treatment by osteotomy of the ulna. Vet Comp Orthop Traumatol. 1995;8:170‐175.

[12] Gnudi G , Martini FM , Zannicheli S , et al. Incomplete humeral condylar fracture in two English Pointer dogs. Vet Comp Orthop Traumatol. 2005;18:243‐245. 16594393

[13] Danielson KC , Fitzpatrick N , Muir P , Manley PA . Histomorphometry of fragmented medial coronoid process in dogs: a comparison of affected and normal coronoid processes. Vet Surg. 2006;35(6):501‐509. 16911150 10.1111/j.1532-950X.2006.00183.x

[14] Proks P , Necas A , Stehlik L , Srnec R , Griffon DJ . Quantification of humeroulnar incongruity in Labrador Retrievers with and without medial coronoid disease. Vet Surg. 2011;40(08):981‐986. 22091872 10.1111/j.1532-950X.2011.00907.x

[15] Samoy Y , Gielen I , Saunders J , van Bree H , Van Ryssen B . Sensitivity and specificity of radiography for detection of elbow incongruity in clinical patients. Vet Radiol Ultrasound. 2012;53(03):236‐244. 22151597 10.1111/j.1740-8261.2011.01900.x

[16] Blond L , Dupuis J , Beauregard G , Breton L , Moreau M . Sensitivity and specificity of radiographic detection of canine elbow incongruence in an in vitro model. Vet Radiol Ultrasound. 2005;46(03):210‐216. 16050278 10.1111/j.1740-8261.2005.00047.x

[17] Gemmill TJ , Hammond G , Mellor D , Sullivan M , Bennett D , Carmichael S . Use of reconstructed computed tomography for the assessment of joint spaces in the canine elbow. J Small Anim Pract. 2006;47(02):66‐74. 16438693 10.1111/j.1748-5827.2006.00052.x

[18] Gemmill TJ , Clements DN . Fragmented coronoid process in the dog: is there a role for incongruency? J Small Anim Pract. 2007;48(07):361‐368. 17490441 10.1111/j.1748-5827.2007.00320.x

[19] Kramer A , Holsworth IG , Wisner ER , Kass PH , Schulz KS . Computed tomographic evaluation of canine radioulnar incongruence in vivo. Vet Surg. 2006;35(01):24‐29. 16409405 10.1111/j.1532-950X.2005.00107.x

[20] House MR , Marino DJ , Lesser ML . Effect of limb position on elbow congruity with CT evaluation. Vet Surg. 2009;38(2):154‐160. 19236672 10.1111/j.1532-950X.2008.00482.x

[21] Werner H , Winkels P , Grevel V , Oechtering G , Böttcher P . Sensitivity and specificity of arthroscopic estimation of positive and negative radio‐ulnar incongruence in dogs. An in vitro study. Vet Comp Orthop Traumatol. 2009;22(06):437‐441. 19876523 10.3415/VCOT-08-12-0125

[22] Wagner K , Griffon DJ , Thomas MW , et al. Radiographic, computed tomographic, and arthroscopic evaluation of experimental radioulnar incongruence in the dog. Vet Surg. 2007;36(07):691‐698. 17894596 10.1111/j.1532-950X.2007.00322.x

[23] Coppieters E , Seghers H , Verhoeven G , et al. Arthroscopic, computed tomography, and radiographic findings in 25 dogs with lameness after arthroscopic treatment of medial coronoid disease. Vet Surg. 2016;45(02):246‐253. 26767932 10.1111/vsu.12443

